# Adipocytokines and autoimmunity

**DOI:** 10.1186/ar3412

**Published:** 2011-09-16

**Authors:** Klaus W Frommer, Elena Neumann, Ulf Müller-Ladner

**Affiliations:** 1 Department of Internal Medicine and Rheumatology, Justus-Liebig University Gießen, Kerckhoff-Klinik, Bad Nauheim, D-61231, Germany

## 

By definition, adipo(cyto)kines are cytokine-like mediators produced mainly by adipose tissue. In the human body, they participate in regulating a variety of physiological functions related to energy metabolism [1] and inflammation [2]. Increased or decreased adipokine levels are associated with autoimmune diseases including diabetes mellitus type 1 (DMT1) [3], rheumatoid arthritis (RA) [4,5], ankylosing spondylitis (AS) [6], systemic sclerosis (SSc) [7], systemic lupus erythematosus (SLE) [8] and Behçet's disease [9-12].

*In vitro *data suggest that adipokines may contribute to the progression of RA as they are potent inducers of proinflammatory cytokines, chemokines and matrix metalloproteinases (MMP) in RA effector cells [13-16].

SSc also appears to be associated with adipokines. Contrary to RA, in which substantial intraarticular degradation of extracellular matrix occurs, SSc is characterized by excessive fibrosis. *In vitro *data and data from animal models of fibrosis point towards a dual role of the adipokine adiponectin as presented by numerous groups at the EULAR congress in London, specifically an antifibrotic effect in later stages of SSc and a profibrotic effect in earlier stages, which appears to be induced by proinflammatory cytokines. Likewise, leptin is involved in the development of liver fibrosis [17-19].

So far, no or only little functional information is available regarding the role of adipokines in other autoimmune diseases. Serum level and clinical correlation analyses, however, suggest an association with adipokines.

In AS, elevated resistin serum levels have been found, while adiponectin levels remained unchanged [6,20]. On the other hand, leptin is discussed controversially in AS. Serum levels were decreased in AS according to two studies [20,21], while they were increased according to another [22]. Also, while correlations of leptin with parameters of inflammation (C-reactive protein, IL-6) and disease activity (Bath Ankylosing Spondylitis Disease Activity Index) have been found by Park *et al. *[22], no such correlations could be found by Toussirout *et al. *[20]. Interestingly, peripheral blood mononuclear cells (PBMC) from AS patients express and secrete more leptin, IL-6 and TNF-α than PBMC from control subjects. Additionally, stimulation of PBMC from AS patients with exogenous leptin led to a significantly increased IL-6 and TNF-α production [23]. Hence, leptin might be involved in the pathogenesis of AS.

In SLE, resistin, for example, has been shown to be associated with general inflammation and bone loss, suggesting a proinflammatory and disease-promoting function [24].

However, the exact role of adipokines especially in SSc, AS and SLE is still unclear and will require further investigation. Also, further research is warranted to show whether adipokines may represent potential therapeutic targets in this diseases.
